# Prevalence and Associated Factors of Traction Alopecia in Women in North Sudan: A Community-Based, Cross-Sectional Study

**DOI:** 10.3390/medicina61020195

**Published:** 2025-01-23

**Authors:** Sama Abdallah, Ahmed A. Hassan, Moteb K. Alotaibi, Ishag Adam

**Affiliations:** 1 Department of Dermatology, Qunfudah General Hospital, Qunfudah 28821, Saudi Arabia; 2 Department of Medicine, Faculty of Medicine, University of Khartoum, Khartoum 11115, Sudan; 3 Department of Dermatology, College of Medicine, Qassim University, Buraidah 51452, Saudi Arabia; 4 Department of Obstetrics and Gynecology, College of Medicine, Qassim University, Buraidah 51452, Saudi Arabia; ia.ahmed@qu.edu.sa

**Keywords:** age, traction alopecia, hairstyle, hair care practice, chemicals

## Abstract

*Background and Objectives:* Traction alopecia is a common type of hair loss that primarily results from prolonged tension in hair follicles. This condition is often associated with certain hairstyles and hair care practices that are prevalent in various cultures, especially in Africa. There have been few studies on this issue in Africa, and none have been conducted in Sudan. Therefore, we aimed to examine the prevalence and associated factors of traction alopecia in women in north Sudan. *Materials and Methods:* A community-based, cross-sectional study was conducted in north Sudan in December 2022. Women’s sociodemographic characteristics were assessed using a questionnaire, and hair and scalp examinations were performed. A multivariate binary analysis was performed. *Results:* A total of 192 women participated in the study, and 48 (25.0%) had traction alopecia. The median age of the women was 42.0 years (interquartile range: 32.0–52.0 years). In a multivariate binary analysis, a family history of women with male pattern baldness or thinning (adjusted odds ratio [AOR] = 2.96, 95% confidence interval [CI] = 1.05–8.37) and the use of hair color or chemicals (AOR = 2.98, 95% CI = 1.30–6.83) were positively associated with traction alopecia. In contrast, increasing age was inversely associated with traction alopecia (AOR = 0.96, 95% CI = 0.93–0.99). The women with traction alopecia showed characteristics such as hair breakage, hair loss with the root attached, scalp tenderness, and trichodynia. *Conclusions:* In north Sudan, one in four women is affected by traction alopecia. Increasing awareness of this condition, providing education on proper hair care methods, and conducting large-scale research are essential steps to prevent its occurrence.

## 1. Introduction

Traction alopecia is a prevalent form of hair loss primarily caused by prolonged tension in hair follicles and often results from certain hairstyles and hair care practices common in various cultures [1,2,3]. In Africa, where cultural practices and hairstyles can lead to an increased incidence of traction alopecia, this condition is particularly prevalent in women and girls [1,2,4]. Research has suggested traction alopecia is a common hair issue in many African countries, particularly among young girls who frequently wear tight hairstyles, such as braids, ponytails, and cornrows [1,2,4]. These styles create continuous tension in hair follicles, leading to hair loss. In Africa, studies have indicated that the prevalence of traction alopecia can be as high as one third of women [1,2]. The frequent study of traction alopecia among females rather than males can be justified by several reasons, including the higher prevalence, cultural significance, and the distinct hair care practices that contribute to traction alopecia among women [1,2,4]. This focus on women allows for a more comprehensive understanding of the issue and the development of targeted interventions to support affected women.

Several factors have been identified as contributing to traction alopecia, such as cultural practices, age, sex, education, lack of awareness, and hair chemical use [1,2,3]. In many African cultures, specific hairstyles are associated with hair loss, including traction alopecia [1,2,4]. Unfortunately, these practices often involve tight styling methods that can result in traction alopecia over time [3]. Traction alopecia predominantly affects younger women, particularly during adolescence, when social and cultural pressures to conform to certain hairstyles are the highest [2,5]. Studies have shown that girls in school settings are particularly vulnerable to this condition [2,5]. There is often a lack of awareness about the risks associated with certain hairstyles. Educational initiatives aimed at informing individuals about safer hair care practices could potentially reduce the incidence of traction alopecia [6]. Increasing awareness and promoting safe hair care practices are essential steps for reducing the incidence of this condition. However, such initiatives require a solid scientific foundation to be successful. Therefore, estimating the prevalence and identifying the potential associated factors at the community level is a proactive step, which is mainly because traction alopecia is treatable and reversible when diagnosed early [2,7].

Studies have begun to address the epidemiology of traction alopecia in African countries such as Egypt and Cameroon [1,2]. However, comprehensive research is still required to examine traction alopecia in women in other African countries, including Sudan. Understanding the cultural, social, and economic factors that affect hair care practices is crucial for developing effective prevention and management strategies, particularly in resource-limited settings such as Sudan.

There have been few studies regarding hair loss in Sudan [8,9], and no studies have investigated traction alopecia in this country. Previous studies have shown a high prevalence of traction alopecia in Africa [1,2]. The prevalence of traction alopecia is 34.5% in Cameroonian women [1], 31.6% of South African women [10], and 31.0% of Egyptian female adolescents [2]. Traction alopecia is a reversible form of hair loss when recognized and managed at early stages [2,7], and it is an under-reported and overlooked hair-loss condition [7,11,12]. To the best of our knowledge, the epidemiology of traction alopecia has not been investigated in Sudan, which represents a gap in the current knowledge. Therefore, this study examined the prevalence and associated factors of traction alopecia in women in Almatamah, River Nile State, north Sudan.

## 2. Materials and Methods

### 2.1. Study Area

River Nile State is located in the northern part of Sudan and is one of the 18 states in Sudan. The total population in River Nile State is 1,120,441, according to the last census in 2008 [13]. River Nile State consists of seven localities, one of which is Almatamah Locality. Almatamah Locality consists of three districts, including the Wad Hamid district.

### 2.2. Study Population and Design

The current community-based cross-sectional study was conducted in December 2022 in two villages in the Wad Hamid district, Almatamah Locality, River Nile State, north Sudan. The Wad Hamid district is a neighbor to Khartoum State, which is approximately 120 km from the capital of Sudan (Khartoum). Two villages were chosen randomly from the Wad Hamid district villages list using systematic sampling. Forty to sixty households from each village were selected according to each village’s population density to obtain the desired sample size. The first member in each household who was approached to participate in the study and who met the study inclusion criteria was selected. If the chosen house was uninhabited or its inhabitants refused to participate in the study, the next house was chosen to meet the target number for the study.

**Figure 1:**
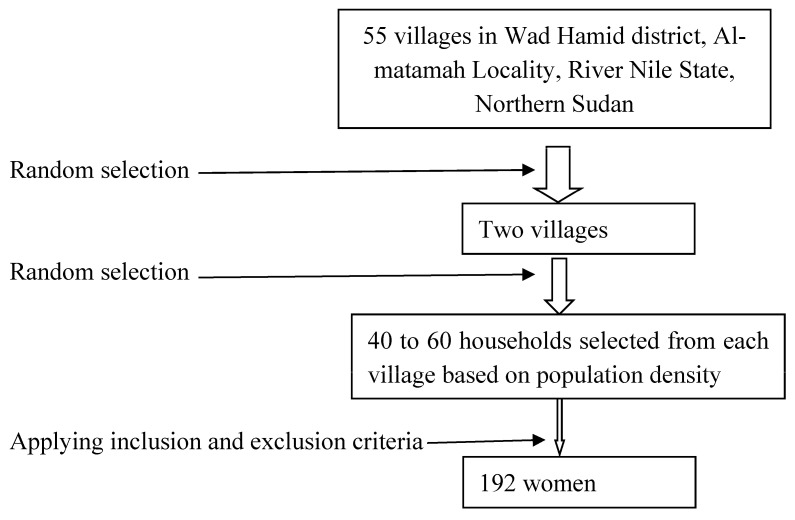
Illustration of the study process.

After giving initial permission to participate in the study and signing an informed consent form, all women aged ≥18 years from the household were enrolled. Women aged <18 years, those with poor cognitive functions, and those who were severely ill were excluded from this study (Figure 1).

### 2.3. Data Collection

This study was conducted according to the Strengthening the Reporting of Observational Studies in Epidemiology (STROBE) guidelines [14]. Data (presence or absence of traction alopecia and possible associated factors) were collected from the participants by administering a questionnaire and conducting a hair and scalp examination for alopecia. The questionnaire was designed to collect relevant information based on previous studies, especially those from an African context [1,2], (Supplementary File). The investigators trained four medical officers (women) with experience in dermatology to carry out the fieldwork. The questionnaire contained information related to the participants’ sociodemographic characteristics, such as age in years, marital status (married/unmarried), educational level (lower than secondary/secondary or higher), occupational status (employed or unemployed), hypertension status (hypertensive/non-hypertensive), and diabetic status (diabetic/non-diabetic). The questionnaire also contained hair-related information, such as a family history of women with male pattern baldness or thinning (yes/no), a family history of men with male pattern baldness or thinning (yes/no), use of color or chemical treatment for hair (yes/no), use of heat treatment for hair (yes/no), wearing of any hair pieces (yes/no), diffuse hair thinning (yes/no), and commonly used hair styling practices (braids, buns or ponytails, and others). An examination of each woman’s hair and scalp was performed, and further information related to the hair and scalp was obtained, such as whether they experienced hair loss in patches, diffuse hair thinning, hair breaking off, hair coming out with the root attached, scalp tenderness, an itchy scalp, a sensitive scalp, a “creepy-crawly” sensation, trichodynia, pain, or a stinging or burning sensation on the scalp, and the duration of hair loss in years. In addition, anthropometric measurements were included (weight and height, expressed as body mass index [BMI] by computing the weight in kg divided by the square of the height in meters [kg/m^2^]). Furthermore, the BMI was subcategorized into normal weight (18.5–24.9 kg/m^2^), underweight (<18.5 kg/m^2^), overweight (25.0–29.9 kg/m^2^), and obese (≥30.0 kg/m^2^) according to the World Health Organization classification [15].

### 2.4. Sample Size Calculation

The sample size was computed using OpenEpi Menu, Version 3. This calculated sample size (192) of women was estimated assuming that one third of the women had traction alopecia, as previously reported in Cameroonian women (34.5%) [1]. We then assumed that 45.0% of the women with traction alopecia used hair chemicals and 30.0% of the women without traction alopecia used hair chemicals. This assumption was based on previous studies [1,2]. This sample size was calculated to detect a difference of 5% at *α* = 0.05 with a power of 80%.

### 2.5. Ethical Statement

The present study was conducted according to the Declaration of Helsinki and good clinical research practices. The study protocol was approved by the Almatamah Health Authority (reference: #9, 2021). All women who participated in this study signed written informed consent forms. The authors followed all measures to ensure the participants’ privacy, confidentiality, and safety (e.g., any personal identifier was excluded at the early data collection stage).

### 2.6. Statistical Analysis

The data were analyzed using IBM Statistical Package for the Social Sciences^®^ (SPSS) for Windows, version 22.0 (IBM Corp., Armonk, NY, USA). Proportions are expressed as percentages. The Kolmogorov–Smirnov test for determining the normality of continuous data, such as age, showed a non-normal distribution. Therefore, age is expressed as the median (interquartile range). A univariate analysis was performed using traction alopecia (for binary regression) as the dependent variable and sociodemographic variables (age, educational level, occupational status, hypertension status, diabetic status, anemia, and BMI) and hair-related variables (family history of women with male pattern baldness or thinning, family history of men with male pattern baldness or thinning, use of color or chemical hair treatment, use of heat treatment for hair, and wearing hair pieces commonly used for hair styling practices) as independent variables. Furthermore, a multivariate binary analysis was performed, including all variables with a *p*-value < 0.20 to control for confounding variables. Variables with few numbers, such as using heat treatment for hair and wearing hair pieces, were excluded from the multivariate analysis. Adjusted odds ratios (AORs) and 95% confidence intervals (CIs) were computed as they were applied. A two-sided *p* value < 0.05 was considered statistically significant.

## 3. Results

Among the 192 women who participated in this study, 48 (25.0%) had traction alopecia, and 144 (75.0%) were unaffected. The median age was 42.0 years (interquartile range: 32.0–52.0). Among the participants, 48 (25.0%) were married, and 144 (75.0%) were unmarried. Regarding education, 142 (74.0%) had secondary education or higher, whereas 50 (26.0%) had lower than secondary education. Additionally, 167 (87.0%) of the women were unemployed, while 25 (13.0%) were employed. Forty-three (22.4%) women had hypertension and 28 (14.6%) had diabetes. Furthermore, 55 (28.6%) participants appeared to be anemic (Table 1).

In the univariate binary analysis, factors positively associated with traction alopecia included a family history of women with male pattern baldness or thinning, a family history of men with male pattern baldness or thinning, use of hair color or chemical treatments, use of heat treatments, and wearing hairpieces. In contrast, age was inversely associated with traction alopecia. Other variables, such as marital status, education, occupation, BMI, anemia, hypertension, and diabetes mellitus, were not associated with traction alopecia (Table 1). In the multivariate binary analysis, a family history of women with male pattern baldness or thinning (AOR = 2.96, 95% CI = 1.05–8.37) and the use of hair color or chemical treatments (AOR = 2.98, 95% CI = 1.30–6.83) were associated with traction alopecia, while age was inversely associated with traction alopecia (AOR = 0.96, 95% CI = 0.93–0.99). A family history of men with male pattern baldness or thinning was identified as a confounding factor (Table 2).

Table 3 shows the common hair and scalp characteristics observed in women with traction alopecia. These characteristics included hair breakage (70.8%), hair loss with the root attached, scalp tenderness (54.2%), and trichodynia (64.6%).

## 4. Discussion

The main findings of this study were that one in four (25%) women in north Sudan had traction alopecia. A younger age and certain hair care practices were associated with traction alopecia in women in north Sudan. This prevalence of traction alopecia is similar to that of previous reports in Africa [1]. This relative prevalence of traction alopecia compared with other African countries (Cameroon and South Africa) could be explained by variations in ethnicity in different regions of Sudan (Arabs predominantly in the northern region).

This study showed that younger age was associated with a high prevalence of traction alopecia. This result supports the assumption of the cultural influence of using unusual hairstyling on the young generation [7,16]. However, this finding is in contrast to a previous study in Cameroon, which showed a positive correlation between increasing women’s age and the occurrence and severity of traction alopecia [1]. In our study, the low occurrence of traction alopecia in older women could be explained by cultural differences (regarding hair care practices) between the two generations (young vs. old). This possibility could explain the recently observed increase in traction alopecia in children and adults [7]. Additionally, our finding could be because of the higher use of chemicals in girls and young women than in older women. Khumalo et al. reported a higher use of hair chemicals in girls than in women (78% vs. 58.7%) [10]. In contrast, increasing age was found to be associated with other types of alopecia, such as female pattern alopecia [17].

In this study, while a family history of women with male pattern baldness was positively associated with traction alopecia, a family history of men with male pattern baldness was a confounding factor. A family history of hair loss was commonly reported with other types of alopecia, such as female pattern alopecia [17,18]. This association between traction alopecia and a family history of women with male pattern baldness opens the door to exploring the possibility of genetic predisposition to traction alopecia. 

In the current study, only a few women used hair heat treatment (*n* = 12), and most (*n* = 10) had traction alopecia. Similarly, several other studies recommended avoiding hair heat treatment to prevent hair damage and traction alopecia in African women [1,19]. Similar to this study, other studies have reported an increase in the risk of traction alopecia for women with traction-based hairstyles, especially when tight hairstyles and the use of hair chemicals coexist [7,16].

Consistent with this study, avoiding the use of hair pieces (nets, caps, and head ties) and using hair chemicals are protective factors against traction alopecia [1,16]. Although few (*n* = 7) women wore hair pieces in this study, they were significantly associated with traction alopecia (5/7 had traction alopecia). Additionally, excessively long hair might contribute to traction alopecia by adding more weight to the hair.

In this study, while improper hair care practices, such as using hair chemicals, were associated with traction alopecia, hairstyling alone was not associated with traction alopecia. In contrast, other studies have reported a positive association between traction alopecia and certain hairstyling practices, such as braids, ponytails, and buns [2,16,20]. This discrepancy between studies may be attributed to the magnitude and duration of tension and the frequency of hairstyling rather than the specific styles themselves. Therefore, irrespective of hairstyling practices, loose hairstyles positioned lower than head level should be chosen, and hairpieces that may increase tension on the hair should be avoided to reduce the risk of traction alopecia. Mirmirani et al. highlighted the importance of communicating information to the community to increase awareness of traction alopecia [21]. They recommended that populations at high risk and hairdressers should limit the duration of traction hairstyles to short periods (i.e., a maximum of two weeks). They advised that these hairstyles should be worn infrequently, painless, and preferably applied to natural hair. Additionally, they suggested incorporating hairdressing education into school curricula to help prevent hair loss [21]. Alhanshali et al. also suggested methods of mitigating the risk of alopecia in people who wear religious head coverings without compromising religious beliefs [3]. However, a study in India showed an incidental diagnosis of traction alopecia, particularly among school girls (tight braids are required as part of their curriculum) [11]. Unlike African contexts, other types of alopecia, such as alopecia areata, are frequently reported in countries like Singapore [12] and the United Kingdom [22]. This difference may be attributed to cultural variations in hair care and styling practices. In the United States, women of African descent who engage in various forms of traumatic hairstyling for extended periods are more susceptible to traction alopecia with approximately one third affected by this condition [16].

In the present study, other factors, such as marital status, education and occupation, hypertension, diabetes mellitus, and anemia, were not associated with traction alopecia. However, diabetes and anemia are related to other types of alopecia, such as female pattern alopecia [18,23]. This lack of association between these factors and traction alopecia supports the assumption that hairstyles and hair care practices are the main factors associated with traction alopecia. However, in our context, the absence of an association between diabetes and traction alopecia may be attributed to the underdiagnosis of diabetes, as indicated by our previous data [24]. This study relied on self-reported diabetes status. Diabetes can significantly influence different types of alopecia, including traction alopecia in women, through several mechanisms that affect hair health and growth. Studies reported that diabetes influences traction alopecia in women through poor circulation, hormonal changes, increased hair follicle sensitivity, and delayed healing, highlighting the importance of managing diabetes to mitigate hair loss risks [25,26].

The findings of this study have important implications for enhancing women’s hair health because traction alopecia is a preventable and treatable condition. Hair health can be improved through various preventive measures, such as promoting a healthy diet, implementing early screening programs, and encouraging lifestyle modifications, including avoiding certain hairstyles and proper hair care practices. The results of the current study will be shared with healthcare professionals and authorities, particularly in Almatamah Locality, to address women’s hair health issues. As a short-term outcome, medical officers advised women with traction alopecia to visit the nearest healthcare facility for further evaluation and management of their hair. In this study, a considerable number (45.8%) of women developed traction alopecia within one year. Balazic et al. showed that the duration of hair loss was not associated with improved loss in patients with traction alopecia at a follow-up visit [6]. However, the ongoing conflict in Sudan poses a major challenge to implementing any advice. A conflict increases mental health disorders and sleep quality, which can exacerbate hair loss, including traction alopecia [27]. Hair loss has a negative effect on mental health disorders (depressive and anxiety disorders), quality of life, social/romantic relationships, familial unit, occupation, productivity, and finances in children and adults [28,29,30,31,32]. Additionally, a conflict can affect the quality and quantity of food, especially in vulnerable populations, such as children, adolescents, and women [33]. As a result, hair loss occurs because of a deficiency of essential nutrients for hair growth [34].

In this study, commonly identified characteristics (>50%) in the hair and scalps of the women with traction alopecia were hair breaking off, hair coming out with the root attached, scalp tenderness, and trichodynia, which can be used as early symptoms/signs for identifying women with traction alopecia. Identifying women who have a greater risk of traction alopecia will facilitate the early detection of this condition and the implementation of preventive and treatment strategies for it. However, further research must confirm and better examine the associations between these hair and scalp characteristics and traction alopecia.

Preventive and treatment strategies for traction alopecia in women focus on minimizing hair stress and promoting healthy hair growth while also considering the potential relationship between diabetes and hair health. Preventive strategies include hairstyle modification such as avoiding tight hairstyles, e.g., braids and ponytails, reducing mechanical stress on hair follicles, and preventing traction alopecia. Opting for looser hairstyles is recommended; nutritional awareness, such as maintaining a balanced diet rich in vitamins and minerals such as iron, is essential for hair health. Women with diabetes should monitor their blood sugar levels, as poor control can negatively impact hair growth. Early identification of alopecia is key to an effective treatment strategy, which involves regular hair checking and seeking health advice from the nearest healthcare facility. By combining these strategies, women can effectively manage traction alopecia while addressing any underlying health concerns related to diabetes.

### Strengths and Limitations of the Study

To our knowledge, this study is the first to examine the epidemiology of traction alopecia in Sudan. Our findings add important knowledge to the limited research on traction alopecia in Africa [1,2,10]. These results can help decision-makers and healthcare professionals enhance women’s hair health because traction alopecia is a preventable, treatable, and reversible condition, particularly when identified in its early stages [2,7]. However, this study has some limitations that should be acknowledged to improve future research designs. This study was conducted in a single region of north Sudan and may not represent the entire country characterized by its multi-ethnic population. Additionally, this study did not include women in urban areas, and different cultures regarding hairdressing and hair care practices may exist between urban and rural communities [2]. Such cultural differences could affect women’s susceptibility to hair loss, particularly traction alopecia. Therefore, a sizable study including different regions of Sudan (urban and rural) is required to quantify the problem of traction alopecia at the national level and to develop a precise preventive strategy. Furthermore, future studies should analyze in more detail the four factors involved in traction alopecia (hairstyle tension, use of chemicals, use of heat, and excessive weight, even including long hair).

## 5. Conclusions

In north Sudan, one in four women is affected by traction alopecia. Factors such as younger age, specific hairstyles, and hair care practices are associated with traction alopecia. Increasing awareness of traction alopecia, educating on proper hair care methods, and conducting large-scale research are essential steps to prevent its occurrence.

## Figures and Tables

**Table 1:** Univariate binary analysis of the factors associated with traction alopecia among women in northern Sudan, 2022 (*n* = 192).

Variables		Total (Number = 192)		Women with Traction Alopecia (Number = 48)		Women Without Traction Alopecia (Number = 144)		Odd Ratio (95% Confidence Interval)	*p* Value
		Median (interquartile range)							
Age, years		42.0 (32.0–52.0)		35.0 (28.0–46.75)		45.0 (35.0–55.0)		0.96 (0.93–0.99)	0.007
		Frequency	Percentage	Frequency	Percentage	Frequency	Percentage		
Marital status	Married	48	25.0	14	29.2	34	23.6	Reference	0.442
	Unmarried	144	75.0	34	70.8	110	76.4	0.75 (0.36–1.56)	
Education level	≥secondary	142	74.0	35	72.9	107	74.3	Reference	0.849
	<secondary	50	26.0	13	27.1	37	25.7	1.07 (0.51–2.25)	
Occupation status	Employed	25	13.0	7	14.6	18	12.5	Reference	0.711
	Unemployed	167	87.0	41	85.4	126	87.5	0.84 (0.32–2.15)	
Hairstyling practices	Buns or ponytails	110	57.3	38	79.2	72	50.0	1.75 (0.45–6.77)	0.412
	Braids	69	35.9	7	14.6	62	43.1	0.37 (0.08–1.70)	0.204
	Others	13	6.8	3	6.2	10	6.9	Reference	
Family history of females with male pattern baldness or thinning	No	165	87.3	36	75.0	129	91.5	Reference	
	Yes	24	12.7	12	25.0	12	8.5	3.58 (1.48–8.65)	0.005
Family history of males with male pattern baldness or thinning	No	115	60.5	21	43.8	94	66.2	Reference	0.007
	Yes	75	39.5	27	56.2	48	33.8	2.52 (1.29–4.91)	
Usage of color or chemical treat for hair	No	154	80.2	30	62.5	124	86.1	Reference	0.001
	Yes	38	19.8	18	37.5	20	13.9	3.72 (1.76–7.88)	
Usage of heat treatment for hair	No	180	93.8	38	79.2	142	98.6	Reference	<0.001
	Yes	12	6.2	10	20.8	2	1.4	18.68 (3.93–88.89)	
Wearing of any hair pieces	No	185	96.4	43	89.6	142	98.6	Reference	0.014
	Yes	7	3.6	5	10.4	2	1.4	8.26 (1.55–44.07)	
Body mass Index groups	Normal	55	28.6	14	29.2	41	28.5	Reference	
	Underweight	10	5.2	3	6.2	7	4.9	1.25 (0.29–5.52)	0.764
	Overweight	63	32.8	14	29.2	49	34.0	0.83 (0.35–1.96)	0.681
	Obese	64	33.3	17	35.4	47	32.6	1.05 (0.46–2.41)	0.891
Anemia	No	137	71.4	33	68.8	104	72.2	Reference	0.645
	Yes	55	28.6	31.2	31.2	40	27.8	1.18 (0.58–2.41)	
Known hypertensive	No	149	77.6	38	79.2	111	77.1	Reference	0.764
	Yes	43	22.4	10	20.8	33	22.9	0.88 (0.39–1.97)	
Known diabetic	No	164	85.4	37	77.1	127	88.2	Reference	0.063
	Yes	28	14.6	11	22.9	17	11.8	2.22 (0.96–5.16)	

**Table 2:** Adjusted multivariate analysis for factors associated with traction alopecia among women in northern Sudan, 2022.

Variables		Odds Ratio	95% Confidence Interval	*p* Value
Age, years		0.96	0.93–0.99	0.013
Family history of females with male pattern baldness or thinning	No	Reference category		0.040
	Yes	2.96	1.05–8.37	
Family history of males with male pattern baldness or thinning	No	Reference category		0.211
	Yes	1.64	0.75–3.60	
Usage of hair color or chemicals	No	Reference category		0.010
	Yes	2.98	1.30–6.83	
Known diabetic	No	Reference category		0.112
	Yes	0.46	0.18–1.19	

**Table 3:** Characteristics of women with traction alopecia (*n* = 48).

Variables		Number	Percentage
Hair loss in patches	No	46	95.8
	Yes	2	4.2
Diffuse hair thinning	No	26	54.2
	Yes	22	45.8
Hair breaking off	No	14	29.2
	Yes	34	70.8
Hair coming out with root attached	No	17	35.4
	Yes	31	64.6
Any scalp tenderness	No	26	54.2
	Yes	22	54.8
Itchy scalp	No	26	54.2
	Yes	22	45.8
Sensitivity scalp	No	40	83.3
	Yes	8	16.7
Creepy-crawly sensation	No	37	77.1
	Yes	11	22.9
Trichodynia	No	17	35.4
	Yes	31	64.6
Feel pain, stinging, or burning sensation on your scalp	No	35	72.9
	Yes	13	27.1
Duration of hair loss, years	≤1	22	45.8
	>1 to ≤5	16	33.3
	>5	10	20.8

## Data Availability

Data can be obtained from corresponding authors upon reasonable request.

## Supplementary Materials

The following supporting information can be downloaded at: https://www.mdpi.com/article/10.3390/medicina61020195/s1, Supplementary File: The questionnaire.
