# Supplementary material for: Prevalence and Associated Factors of Traction Alopecia in Women in North Sudan: A Community-Based, Cross-Sectional Study
Source: Medicina (Kaunas). 2025 Jan 23;61(2):195. doi: 10.3390/medicina61020195 (PMC11857667; doi:10.3390/medicina61020195)
Supplement: Supplementary file 1 [file medicina-61-00195-s001.zip › medicina-3400133-supplementary.pdf]

Supplementary file: The questionnaire

Prevalence and associated factors of traction alopecia in women in North Sudan: A community-based, cross-sectional study

Section 1

Sociodemographic data and clinical profile

1. Women age in years…..
2. Marital status a. Married b. Unmarried
3. Education level: a. ≥ secondary b. < secondary
4. Occupation status a. Employed b. Unemployed
5. Family history of females with male pattern baldness or thinning: a. No b. Yes
6. Family history of males with male pattern baldness or thinning: a. No b. Yes
7. Known hypertensive a. No b. Yes
8. Known diabetic a. No b. Yes

Section 2 Hair care practices

1. Hairstyling practices a. Buns or ponytails b. Braids c. Others please specify…..
2.

	No	Yes
3. Usage of color or chemical treat for hair:		
4. Usage of heat treatment for hair		
5. Wearing of any hair pieces:		

Section 3 Characteristics of women with traction alopecia

1.		No	Yes
2.	Hair loss in patches		
3.	Diffuse hair thinning		
4.	Hair breaking off		
5.	Hair coming out with root attached		
6.	Any scalp tenderness		
7.	Itchy scalp		
8.	Sensitivity scalp		
9.	Creepy-crawly sensation		
10.	Trichodynia		
11.	Feel pain, stinging, or burning sensation on your scalp		
12.	Duration of hair loss, years		

Section 3

Physical examination and anthropometric measurements

1. On examination, the women look anemic a. No b. Yes
2. Women heigh in cm …………………………………….
3. Women weight in kg ………………………………………..

Many thanks for your particpation
